# PCSK9 inhibition: the dawn of a new age in cholesterol lowering?

**DOI:** 10.1007/s00125-016-4178-y

**Published:** 2016-12-26

**Authors:** David Preiss, Marion Mafham

**Affiliations:** Medical Research Council Population Health Research Unit, Clinical Trial Service Unit and Epidemiological Studies Unit, Nuffield Department of Population Health, University of Oxford, Richard Doll Building, Old Road Campus, Roosevelt Drive, Oxford, OX3 7LF UK

**Keywords:** Adnectin, Cardiovascular, Clinical trial, Monoclonal antibody, PCSK9, Review, siRNA

## Abstract

Proprotein convertase subtilisin/kexin type 9 (PCSK9) is a circulating enzyme of hepatic origin that plays a key role in LDL receptor turnover. Genetic studies have confirmed that individuals with gain-of-function *PCSK9* mutations have increased PCSK9 activity, elevated LDL-cholesterol levels and a severe form of familial hypercholesterolaemia. Those with variants leading to reduced PCSK9 have lower LDL-cholesterol levels and a reduced risk of coronary heart disease, and this has led to the development of various strategies aimed at reducing circulating PCSK9. Monoclonal antibodies to PCSK9, given every 2–4 weeks by subcutaneous injection, have been shown to reduce LDL-cholesterol by 50–60% compared with placebo in individuals with and without diabetes. PCSK9 inhibition also reduces lipoprotein(a), an atherogenic lipid particle, by around 20–30%. Major cardiovascular outcome trials for two agents, evolocumab and alirocumab, are expected to report from 2017. These trials involve over 45,000 participants and are likely to include about 15,000 individuals with diabetes. PCSK9-binding adnectins have been employed as an alternative method of removing circulating PCSK9. Small interfering RNA targeting messenger RNA for PCSK9, which acts by reducing hepatic production of PCSK9, is also under investigation. These agents may only need to be given by subcutaneous injection once every 4–6 months. Ongoing trials will determine whether anti-PCSK9 antibody therapy safely reduces cardiovascular risk, although high cost may limit its use. Development of PCSK9-lowering technologies cheaper than monoclonal antibodies will be necessary for large numbers of individuals to benefit from this approach to lowering cholesterol.

## Introduction

Proprotein convertase subtilisin/kexin type 9 (PCSK9) inhibitors are powerful LDL-cholesterol-lowering medications currently being investigated in major cardiovascular outcome trials. In this review, we discuss the following topics: the biology of PCSK9; studies confirming the causal relationship between PCSK9 and cardiovascular disease (CVD); the various classes of PCSK9 inhibitors at different stages of investigation; the efficacy (in people with and without diabetes where data are available) and safety of PCSK9 inhibitors and their potential position in clinical practice.

## Biology of PCSK9

Hepatic expression of the LDL receptor is a major determinant of circulating LDL-cholesterol [1]. Individuals with heterozygous familial hypercholesterolaemia (FH) exhibit either reduced expression of LDL receptors or reduced binding of apolipoprotein B to the LDL receptors, with a resultant increase in circulating LDL-cholesterol and elevated cardiovascular risk [2]. Statins increase hepatic LDL receptor expression, consequent to low intracellular cholesterol levels. The resulting enhanced uptake of LDL from the circulation and reduction in blood LDL-cholesterol concentration reduces cardiovascular risk in people with and without diabetes [3, 4].

**Fig. 1:**
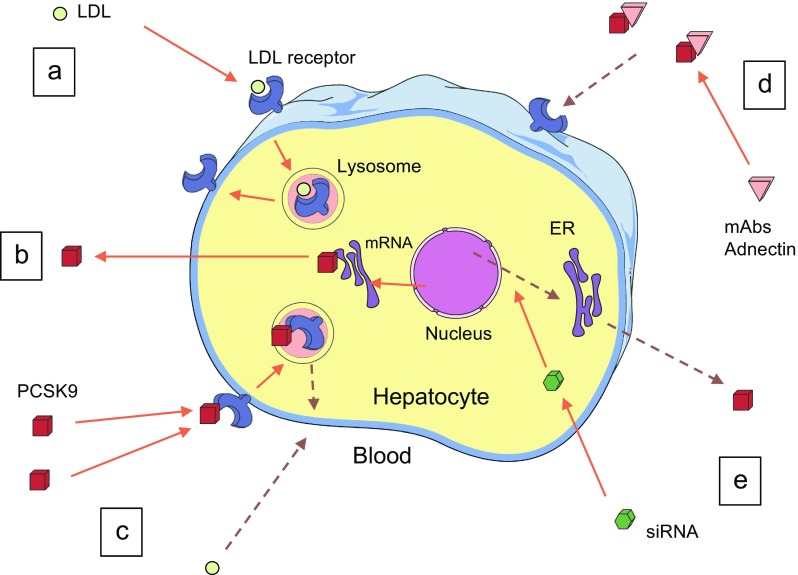
The effect of PCSK9 on LDL receptor turnover and mechanisms of action of different PCSK9 inhibitors. (**a**) In the absence of PCSK9, the LDL receptor is recirculated to the cell surface after carrying LDL into lysosomes. (**b**) PCSK9 is produced by the liver and enters the circulation. (**c**) When PCSK9 binds to the LDL receptor, it undergoes lysosomal degradation and is not recirculated to the cell surface, thereby preventing uptake of LDL (dashed lines). (**d**) Monoclonal antibodies to PCSK9 and adnectins act by binding to PCSK9, thereby removing it from the circulation and preventing binding of PCSK9 to the LDL receptor (dashed line). (**e**) siRNAs act by degrading mRNA, thereby reducing PCSK9 release to the circulation (dashed line). ER, endoplasmic reticulum; mAb, monoclonal antibody

PCSK9 belongs to a family of proteases called proprotein convertases, which catalyse the conversion of secretory precursors into active products [5]. It is a circulating protein of hepatic origin, expressed from a genetic locus on chromosome arm 1p32.3, which is intimately involved in hepatic LDL receptor turnover [6]. Under normal conditions, when PCSK9 binds to the LDL receptor and is internalised, lysosomal degradation follows and there is no recirculation of that LDL receptor to the hepatocyte surface (Fig. 1). Thus, PCSK9 reduces LDL receptor expression by the liver, resulting in reduced uptake of LDL from the circulation and, consequently, higher circulating LDL-cholesterol levels [7].

The potential importance of PCSK9 to LDL-cholesterol homeostasis and cardiovascular risk was identified in various seminal genetic studies. In 2003, Abifadel and colleagues reported that two kindreds with premature CVD and apparently unexplained autosomal dominant hypercholesterolaemia, based on known FH genetic mutations, were affected by gain-of-function mutations in the *PCSK9* gene leading to increased activity of PCSK9 and marked hypercholesterolaemia [8]. PCSK9 activity has since been confirmed as a key determinant of LDL-cholesterol levels and mutations in *PCSK9* have been confirmed as the cause of a very rare, but particularly severe, form of FH. Complementary information came from studies of individuals with loss-of-function *PCSK9* mutations and low PCSK9 activity. In the Atherosclerosis Risk in Communities Study, about 1 in 40 black participants (*n* = 3363) carried a nonsense mutation (*PCSK9* ^142X^ or *PCSK9* ^679X^) in the *PCSK9* gene [9]. This genotype was associated with 28% lower LDL-cholesterol levels and a HR for CHD of 0.11 (95% CI 0.02, 0.81), admittedly with wide CIs given the small number of coronary events. Similarly, of 9524 white individuals, about 1 in 30 had a heterozygous sequence variation (*PCSK9* ^46L^) in *PCSK9* that was associated with 15% lower LDL-cholesterol levels and a halving in the risk of CHD (adjusted HR 0.50; 95% CI 0.32, 0.79). These findings have subsequently been replicated in larger studies [10]. Furthermore, individuals with compound heterozygous loss-of-function mutations in *PCSK9* and, consequently, no circulating PCSK9 and very low LDL-cholesterol levels appear to suffer no obvious clinical disadvantage from the lack of PCSK9 [11].

## Available inhibitors of PCSK9

The aim of therapy with PCSK9 inhibitors is to reduce circulating PCSK9 levels, thereby increasing hepatic LDL receptor expression and lowering circulating LDL-cholesterol levels. Current therapeutic approaches involve either the binding of circulating PCSK9 (monoclonal antibodies and modified binding proteins) or reducing the hepatic production of the protein (small interfering RNAs [siRNAs]) (Fig. 1). Therapies currently under investigation are given by subcutaneous injection; we are not aware of any ongoing clinical trials of an oral PCSK9-lowering preparation, although attempts have been made to develop orally administered agents.

### Monoclonal antibodies to PCSK9

Monoclonal antibodies targeted at PCSK9 are the best-developed strategy for lowering PCSK9. Two agents, evolocumab and alirocumab, are currently under investigation in major Phase 3 clinical trial programmes (Table 1) [12, 13]. Data for bococizumab are also included in Table 1 despite the recent discontinuation of clinical trials with this agent due to attenuation of LDL-cholesterol lowering over time and comparatively higher levels of immunogenicity and greater frequency of injection-site reactions [14, 15]. Evolocumab and alirocumab are fully human antibodies while bococizumab is a partially humanised antibody. Notably, at least one-third of participants in the major trials have diabetes. The first of these trials is expected to report in 2017. Research programmes for some PCSK9 monoclonal antibodies have been discontinued in Phase 2 (e.g. RG-7652) while others have yet to enter Phase 3 (e.g. LY3015014) [16]. These agents are given by subcutaneous injection, typically once every 2–4 weeks [12, 13, 15] although some are now being tested at intervals of 8 weeks [16]. Patients require training on how to administer the therapy. The drug needs to be stored in a refrigerator and then warmed to room temperature before injection.

**Table 1:** Placebo-controlled cardiovascular outcome trials of PCSK9 inhibitors

Study	Inhibitor	*N*	% with diabetes	Intervention	Population	Planned follow-up at start of trial	Primary outcome	Expected year of completion
FOURIER (TIMI-59) [12], Clinical.trials.gov NCT01764633	Evolocumab	27,564	∼34	Evolocumab 140 mg every 2 weeks or 420 mg monthly or placebo	Prior vascular event; LDL-cholesterol ≥1.8 mmol/l or non–HDL-cholesterol ≥2.6 mmol/l	∼4 years	Cardiovascular death, MI, stroke, coronary revascularisation	2017
ODYSSEY OUTCOMES [13], Clinical.trials.gov NCT01663402	Alirocumab	18,000	Not available	Alirocumab 75 mg or 150 mg every 2 weeks; or placebo	ACS in last 12 months; LDL-cholesterol ≥1.8 mmol/l or non-HDL-cholesterol ≥2.6 mmol/l, or apolipoprotein B ≥0.8 g/l	Until >1613 events occur and >2 years follow-up	Coronary events or ischaemic stroke	2018
SPIRE-1 [15], Clinical.trials.gov NCT01975376	Bococizumab^a^	17,000	∼40	Bococizumab 150 mg every 2 weeks; or placebo	High risk or prior vascular event; LDL-cholesterol 1.8–2.5 mmol/l or non-HDL-cholesterol 2.6–3.3 mmol/l	∼5 years	Cardiovascular death, MI, stroke, urgent coronary revascularisation	–
SPIRE-2 [15], Clinical.trials.gov NCT01975389		9000		Bococizumab 150 mg every 2 weeks; or placebo	High risk or prior vascular event, LDL-cholesterol ≥2.6 mmol/l or non-HDL-cholesterol ≥3.4 mmol/l	∼4 years	Cardiovascular death, MI, stroke, urgent coronary revascularisation	–

All drugs are administered by subcutaneous injection

^a^The development of bococizumab was recently discontinued and the SPIRE trials (both fully enrolled) have been stopped [14]

ACS, acute coronary syndrome; MI, myocardial infarction

### Modified binding-protein inhibitors of PCSK9 (adnectins)

While the inhibition of PCSK9 by small molecules has proven challenging, PCSK9-binding adnectin-based polypeptides have been developed [17]. Adnectins are compact proteins with branched-out loops, derived from human fibronectin tenth type III domain, which can be modified to bind a target with strong affinity. Data are available from Phase 1 trials in 64 healthy participants to whom single ascending doses of BMS-962476 were given intravenously or subcutaneously and participants were followed for up to 6 weeks [18]. LDL-cholesterol reductions of up to 48% occurred between days 4 and 14 post dose. There are no ongoing clinical trials of this agent at present.

### Targeting PCSK9 with siRNA

siRNA has been developed which acts within the hepatocyte to inhibit PCSK9 synthesis by degrading the mRNA of the intended protein [19]. In a Phase 1 single-escalating-dose placebo-controlled clinical trial conducted in 32 healthy volunteers with LDL-cholesterol ≥3.0 mmol/l, ALN-PCS was given intravenously [20]. The highest dose studied (0.4 mg/kg) lowered LDL-cholesterol by 40% on average. In subsequent Phase 1 studies various doses, both single and multiple, have been given subcutaneously. A potential benefit of this agent’s mechanism of action lies in its prolonged effect on LDL-cholesterol. Single doses of 300 mg and 500 mg lowered LDL-cholesterol levels by about 50% at 12 weeks [21]. This difference, when compared with placebo, was largely preserved (at approximately 40%) at 6 months post dose, suggesting that a dose need only be administered once every 4–6 months. Separate results for individuals with diabetes are not yet available.

## Effects of monoclonal antibodies to PCSK9 in people with and without diabetes

Monoclonal antibodies to PCSK9 have typically yielded reductions in LDL-cholesterol levels of 50–60% compared with placebo at the doses tested in the major Phase 3 trials [22]. Table 2 provides examples of the lipid-modifying effects of evolocumab, alirocumab and bococizumab from selected trials. Maximal reduction of LDL-cholesterol levels is achieved when all PCSK9 is bound and, therefore, further increases in drug dose have no further effects (apart from modestly prolonging the duration of action). Unlike treatment with statins, which vary in LDL-cholesterol-lowering efficacy, these PCSK9 inhibitors are expected to provide similar maximal effect. In addition, it is well recognised that PCSK9 inhibition leads to a reduction in levels of lipoprotein(a), an atherogenic lipid particle causally implicated in CVD [23], of about 20–30% [24, 25]. Statins have no such effect. The reduction in lipoprotein(a) levels is not fully understood and kinetic lipid studies are required to elucidate the mechanism behind this.

**Table 2:** Effects of monoclonal antibodies to PCSK9 on circulating lipids–results from selected placebo-controlled trials

Monoclonal antibody	Trial	Population	*N*	Duration (weeks)	Dose	LDL-cholesterol	Total cholesterol	HDL-cholesterol	Triacylglycerols	Lp(a)
Evolocumab	DESCARTES [38], Clinical.trials.gov NCT01516879	LDL-cholesterol ≥1.9 mmol (with or without atorvastatin depending on baseline risk)	901	52	420 mg every 4 weeks; or placebo	−57 (2)^a^	−34 (1)^a^	+5 (1)^a^	−12 (3)^a^	−22 (2)^a^
Alirocumab	ODYSSEY-LT [36], Clinical.trials.gov NCT01507831	Elevated cardiovascular risk on statin with LDL-cholesterol ≥1.8 mmol/l	2341	24	15 mg every 2 weeks; or placebo	−62 (−64, −59)	−38 (−39, −36)	+5 (+3, +6)	−17 (−20, −15)	−26 (−28, −23)
Bococizumab	Clinical.trials.gov NCT0159224 [30]	Adults on stable statin with LDL-cholesterol ≥2.1 mmol/l	93	24	150 mg every 2 weeks; or placebo	−53 (−63, −43)	−30 (−37, −24)	+1 (−4, +7)	−19 (−35, +5.0)^b^	−9 (−27, +3)^b^
		Adults on stable statin with LDL-cholesterol ≥2.1 mmol/l	97	24	300 mg every 4 weeks; or placebo	−41 (−52, −30)	−24 (−31, −17)	+7 (0, +13)	−14 (−33, +11)^b^	−11 (−27, 0)^b^

Results shown as per cent mean change relative to the placebo group (95% CIs) except where indicated

All drugs were administered by subcutaneous injection

^a^Per cent mean difference (SE)

^b^Per cent median (Q1, Q3) change from baseline on bococizumab, not adjusted for placebo

Lp(a), lipoprotein(a); ODYSSEY-LT, ODYSSEY LONG TERM

See the Text box for a summary of the effects of antibodies to PCSK9.
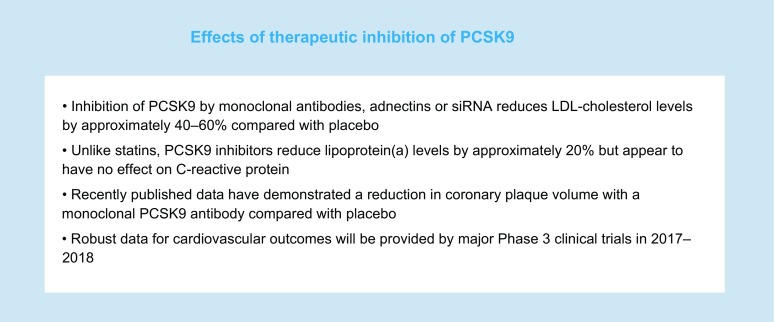


### Lipids and C-reactive protein

Reductions in atherogenic lipids have been similar in trial participants with and without diabetes in whom circulating PCSK9 levels are also similar. A pooled analysis of data from three 12 week evolocumab trials with 2532 participants (413 with diabetes, 2119 without diabetes) was published recently [26]. In individuals with and without diabetes, respectively, when compared with placebo, evolocumab reduced levels of LDL-cholesterol by 60% and 66%, non-HDL-cholesterol by 55% and 58%, total cholesterol by 38% and 40%, triacylglycerols by 23% and 17% and lipoprotein(a) by 31% and 29%. HDL-cholesterol levels rose by 8% and 7% in participants with and without diabetes, respectively. There was no evidence of heterogeneity between diabetic and non-diabetic participants [26]. Furthermore, in a post hoc analysis of the DESCARTES trial, the effect of evolocumab (vs placebo) on LDL-cholesterol at 52 weeks was similar among participants with diabetes (*n* = 120; 51% reduction), impaired fasting glucose (*n* = 293; 59% reduction), the metabolic syndrome (*n* = 289; 55% reduction) and those without these conditions (393; 58% reduction) [27]. Similarly, the 1 year results of two controlled open-label extension trials of evolocumab (vs standard care) showed that the reduction in LDL-cholesterol and triacylglycerol levels was comparable in those with and without diabetes [28].

For alirocumab, 24 week data are available from the Long-term Safety and Tolerability of Alirocumab in High Cardiovascular Risk Patients with Hypercholesterolemia Not Adequately Controlled with Their Lipid Modifying Therapy (ODYSSEY LONG TERM) trial [29]. Participants (2341 participants: 832 with diabetes, 1509 without diabetes) at elevated cardiovascular risk were randomised to receive alirocumab or placebo. Changes in LDL-cholesterol (59% vs 63% reduction in those with vs without diabetes, respectively), triacylglycerol (19% vs 17% reduction) and HDL-cholesterol (3% vs 5% increase) levels, compared with placebo, were similar in those with and without diabetes. We are not aware of data for participants with diabetes from the bococizumab programme [30].

One feature of statin therapy is that while starting doses typically yield relatively large reductions in LDL-cholesterol (e.g. 22%, 32%, 40% and 49% for a 10 mg daily dose of pravastatin, simvastatin, atorvastatin and rosuvastatin, respectively [12]), doubling the dose only provides a further 6% reduction in LDL-cholesterol on average, apparently driven by increased production of PCSK9 in response to statin therapy [13]. It is apparent that the introduction of PCSK9-lowering treatment provides similar, or probably greater, proportional reductions in LDL-cholesterol levels when an individual is on a statin compared with no statin therapy [30].

An interesting finding in these Phase 2 studies has been that although statins moderately lower C-reactive protein levels and have a modest anti-inflammatory effect [31, 32], PCSK9 inhibitors appear to have no effect on C-reactive protein [33].

### Cardiovascular outcomes

Conclusive data regarding the effect of monoclonal antibodies on cardiovascular events are expected from the major outcome trials (Table 1) [12, 13]. Given the well-established effect of PCSK9 inhibitors on LDL-cholesterol and the confirmed causal relationship between LDL-cholesterol levels and CVD [34], results from these major cardiovascular outcome trials are among the most eagerly awaited in the field of cardiovascular and metabolic medicine. While preliminary data from smaller, shorter trials have suggested substantial cardiovascular benefit (as much as a halving of major cardiovascular events) [35, 36], these results are based on only around 130 cardiovascular events, indicating that they should be interpreted with caution. Recently, the GLobal Assessment of Plaque reGression with a PCSK9 antibOdy as Measured by intraVascular Ultrasound (GLAGOV) trial has shown that evolocumab reduced coronary plaque volume (vs placebo) when added to statin therapy. [37].

## Safety profile of monoclonal antibodies to PCSK9

The clinical trial programmes for evolocumab and alirocumab have raised few concerns thus far and adherence to randomised active treatment and placebo has been similar. As noted above, individuals with no measurable circulating PCSK9 and low LDL-cholesterol levels appear to suffer no clinical disadvantage [11]. Putative side effects and the available evidence are summarised below.

### Injection-site reactions

A reaction at the site of injection has typically been reported in around 5% of trial participants, with little difference between placebo- and PCSK9-inhibitor-treated patients [36, 38].

### Anti-drug antibodies

Anti-drug antibodies have not been detected in evolocumab-treated patients [39] and have been found in only a small number of alirocumab-treated patients [40], with no apparent detrimental effect on the LDL-cholesterol-lowering efficacy of the drug. Development of such antibodies may explain the diminishing LDL-cholesterol-lowering effect seen with bococizumab that led to the termination of its development [14].

### Neurocognitive events

In 2014, the US Food and Drug Administration (FDA) mandated that ongoing cardiovascular outcome trials of PCSK9 inhibitors must include detailed evaluation of neurocognitive events, at least in a subset of participants. Two studies (two pooled open-label extension trials of evolocumab; one placebo-controlled trial of alirocumab) showed that a greater number of participants receiving a PCSK9 inhibitor reported neurocognitive adverse events than control participants [35, 36]. However, the open-label design of the evolocumab studies makes this finding difficult to interpret and the blinded alirocumab study included only 22 patients with neurocognitive adverse events. More information will come from the EBBINGHAUS trial, a substudy within the Further Cardiovascular Outcomes Research with PCSK9 Inhibition in Subjects with Elevated Risk (FOURIER) trial, in which measures of cognitive function are the primary outcome [41]. Importantly, lowering LDL-cholesterol by 1–1.5 mmol/l in the statin trials is not associated with any deterioration in measures of cognition [42].

### New-onset diabetes and glycaemic control

Statins appear to have a small diabetogenic effect and low LDL-cholesterol levels may be causally linked to higher diabetes risk [34, 43]. This prompted the listing of new-onset diabetes as a pre-specified outcome in major trials of PCSK9 inhibitors. The first major genetic polymorphism study of *PCSK9* variants and their association with diabetes has suggested that diabetes risk is 15–20% higher in the context of a 1 mmol/l genetically predicted reduction in LDL-cholesterol [44]. However, data from evolocumab and alirocumab clinical trials appear reassuring thus far [27, 45]: although based on small numbers of new-onset diabetes events, even if a diabetogenic effect is found it is likely to be small.

### Very low concentrations of LDL-cholesterol

The combination of intensive statin therapy and PCSK9 inhibition (as used in the cardiovascular endpoint trials) will achieve lower LDL-cholesterol concentrations than have previously been possible. Dose-reduction strategies to address this concern have been tried in participants whose LDL-cholesterol falls below predefined thresholds (with similar steps being taken in an equal number of placebo-treated patients to maintain blinding). The knowledge that free-living individuals with genetically determined low or very low levels of LDL-cholesterol, based on *PCSK9* mutations, appear to suffer no clinical disadvantage [11, 46] is reassuring but data from large-scale trials are required to address this theoretical concern.

## PCSK9 inhibitors: their potential place in clinical practice

Statins are cheap, safe and effective [4] and are likely to remain the mainstay of lipid-lowering treatment for individuals with and without diabetes, particularly the 300 million diabetic individuals in low- or middle-income countries. Currently, evolocumab and alirocumab are both approved by the FDA and the European Medicines Agency for use in some people with FH and in individuals with pre-existing CVD who are on maximal-tolerated statin dose and who require additional lipid-lowering therapy. Recently, an American College of Cardiologists (ACC) Expert Consensus Decision Pathway recommended consideration of PCSK9 inhibitors, after maximising statin therapy and attention to adherence, in several high-risk groups including those with prior CVD and diabetes who have not achieved a ≥50% reduction in LDL-cholesterol and possibly in such individuals who have not achieved a non-HDL-cholesterol level of <2.6 mmol/l [47]. However, recommendations for their use in many countries will require Phase 3 studies to demonstrate not only that these agents are efficacious and safe but also that they are cost effective. Table 3 illustrates the likely absolute benefit that would be expected with PCSK9 inhibitors, assuming that the reduction in cardiovascular events achieved for each mmol/l reduction in LDL-cholesterol is the same as that observed with statins [4]. In the majority of individuals already on statin therapy, the absolute reduction in LDL-cholesterol with a PCSK9 inhibitor will be moderate, at around 1 mmol/l. This would be expected to result in a reduction in major vascular events of around one-quarter. Whether this potential benefit outweighs the cost, treatment burden and any risks for people with diabetes will depend heavily on the cardiovascular risk of an individual. In the UK, rates of hospital admission for CVD (based on a broad definition of International Classification of Diseases coding) in people with diabetes, but no prior CVD, are around 1.5–2.5% per year depending on ethnicity [48], and these rates continue to fall [49]. For primary prevention of CVD in people with diabetes receiving a statin, the absolute benefit of PCSK9 inhibitors is estimated to be around 13–19 people who will avoid major cardiovascular events (per 1000 people treated for 5 years, assuming an annual event rate of 1–1.5%; Table 3). This benefit would be highly unlikely to justify the substantial cost (approximately $14,000 per year in the USA and £4000 per year in the UK) and inconvenience of treatment.

**Table 3:** Putative effects of PCSK9 inhibition on major cardiovascular events in hypothetical patients with different levels of LDL-cholesterol and at different levels of cardiovascular risk

Characteristic	Moderate risk^a^		High risk^b^	
On statin?	Yes	No	Yes	No
Baseline LDL-cholesterol (mmol/l)^c^	2	4	2	4
Reduction in LDL-cholesterol (mmol/l) with PCSK9 inhibitor	1	2	1	2
HR (excluding first year)^d^	0.75	0.56	0.75	0.56
Individuals protected from a cardiovascular event (no. per 1000 treated for 5 years)	13–19	22–33	38–50	66–88

^a^Patients at moderate risk (e.g. those with diabetes but no prior vascular disease): assumed 10 year cardiovascular risk of 10–15%

^b^Patients at high risk (e.g. patient with diabetes and prior vascular disease): assumed 10 year cardiovascular risk of 30–40%

^c^Mean LDL-cholesterol concentration prior to initiation of the PCSK9 inhibitor assumed to be 4 mmol/l in individuals not on a statin and 2 mmol/l for those receiving intensive statin treatment

^d^Effect on cardiovascular events (including myocardial infarction, stroke and vascular death) after the first year of treatment, based on 50% LDL-cholesterol reduction alone (calculated as 0.75^LDL-c reduction [mmol/l]^) [42]

As outlined in the ACC guidelines mentioned above, the likely role for monoclonal antibody PCSK9 inhibitors will, apart from for patients with FH, be in secondary prevention particularly among those at highest risk such as those with diabetes in whom the annual cardiovascular event rate might be around 3–4% per year [50]. In such individuals, the addition of a PCSK9 inhibitor to statin treatment might result in around 38–50 people avoiding suffering a cardiovascular event for every 1000 people treated for 5 years (Table 3). However, at current UK prices this would still cost almost £400,000 for each person protected from suffering a major cardiovascular event (not taking into account the prevention of recurrent CVD events or resulting heart failure). The challenge for the companies developing anti-PCSK9 antibodies and other PCSK9 inhibitors is therefore to produce effective agents at lower cost.

## Conclusions

The results from the keenly awaited FOURIER and ODYSSEY Outcomes trials will soon establish whether lowering LDL-cholesterol via inhibition of PCSK9 produces the expected reductions in major cardiovascular events. Equally important, however, will be establishing whether these treatments are associated with any hazard and identifying populations at sufficiently high vascular risk for this treatment to be cost effective.

## Abbreviations

ACC: American College of Cardiology
CVD: Cardiovascular disease
FDA: Food and Drug Administration
FH: Familial hypercholesterolaemia
FOURIER: Further Cardiovascular Outcomes Research with PCSK9 Inhibition in Subjects with Elevated Risk
PCSK9: Proprotein convertase subtilisin/kexin type 9
siRNA: Small interfering RNA
SPIRE: Studies of PCSK9 Inhibition and the Reduction of Vascular Events
